# Lean on Me: A Scoping Review of the Essence of Workplace Support Among Child Welfare Workers

**DOI:** 10.3389/fpsyg.2020.00287

**Published:** 2020-02-25

**Authors:** Oyeniyi Samuel Olaniyan, Hilde Hetland, Sigurd William Hystad, Anette Christine Iversen, Gaby Ortiz-Barreda

**Affiliations:** ^1^Department of Health Promotion and Development, University of Bergen, Bergen, Norway; ^2^Department of Psychosocial Science, University of Bergen, Bergen, Norway; ^3^Public Health Research Group, University of Alicante, Alicante, Spain

**Keywords:** workplace support, social support, coworker support, leadership/supervisor support, psychosocial risk

## Abstract

Child welfare workers (CWWs) often work under conditions similar in nature to workers within safety critical organizations (SCOs). This is because most of their work surrounds child neglect, securing homes for foster children, haphazard, and intricate cases, among other things, and where making wrong decisions, inattention to details, and the likes could lead to adverse consequences especially for the kids within their care. Research has shown that employees who experience support at work often report less stress symptoms, burnout, and a host of other negative workplace experiences. Experience of support at work has also been found to boost employees’ retention, job satisfaction, and productivity. Despite this development, research exploring the essence of workplace support among CWW is very scarce in the literature, and we know very little about the type of workplace support and their influence on a host of workplace outcomes, especially the negative ones like secondary traumatic stress, aggression, and violence toward CWWs. The purpose of the current scoping review was to uncover what is known about workplace support and their relationship with workplace outcomes among CWWs. The authors explored four databases and identified 55 primary studies investigating workplace support and workplace outcomes among CWWs in the review. Studies mostly framed support under three main support types of coworker/peer support, social/organizational/management support, and supervisor/leadership support. Findings showed that workplace support has a positive impact on workplace variables like job satisfaction, engagement, commitment, and reduces the risk of turnover, burnout, and other negative workplace variables. The review highlights possible directions for future research.

## Introduction

In Norway, a huge number of child welfare workers (CWWs) recently took to the streets with slogans like “HeiErna” (Erna refers to the prime minister of Norway, Erna Solberg). These employees demonstrated to protest the shortage of employees amidst the ever-increasing workloads they face from day to day. According to one of the protesters, the Norwegian government’s promises to increase competences and learning among this workgroup will fail if there are insufficient employees, creating heavy workloads and demands (as cited in www.frifagbevegelsen.no). We know from past research within this workgroup that employees are often confronted with heavy workloads along with other types of unsuitable workplace events.

The purpose of this scoping review is to assess the essence of workplace support especially among CWWs vis-à-vis their constant exposure to risks at work. Owing to the nature of their work (child neglect, securing homes for foster children, haphazard, and intricate cases, among other things), CWWs often find themselves within zones bearing resemblance to employees within safety critical organizations (SCOs). This implies that wrong decisions, inattention to details, and the likes could lead to adverse consequences especially for the kids within their care. Risks at work have been associated with stress, disrupted productivity, sickness, and other negative health outcomes (Cox, 1993; Griffths, 1998; Cox et al., 2000, 2007; Leka et al., 2007). Working around children with troubled pasts, vulnerabilities, and complicated upbringing, CWWs deal with innumerable demanding intricacies within their field. Past research has shown that child welfare is one of the most complex, stressful, and emotionally demanding within the field of human services (Madden et al., 2014; McFadden et al., 2015). Across countries there are concerns that many social workers leave their jobs in child welfare services. Vacancies and a constant stream of new employees have negative effects both on the remaining staff of social workers and the clients by creating instability in services for vulnerable children and families. We know from past research that several workplace resources like job satisfaction, engagement, and social support provide ameliorating effects on employees’ experiences of workplace risks like turnover and burnout. For instance, social support has been found to reduce the risk of turnover, burnout, and other debilitating workplace risks (Karasek and Theorell, 1990; Brough and Pears, 2004; DePanfilis and Zlotnik, 2008). In this regard, getting an overview of the roles of workplace support for this work group will contribute immensely to enhancing and boosting their performance and well-being.

A review of the literature shows a seemingly one-sided focus on negative events or workplace variables like turnover and turnover intentions among CWWs. Only very few studies have explored novel topics that are not only related to turnover/turnover intentions (Jacquet et al., 2008; Hwang and Hopkins, 2012; Lizano et al., 2014; Kim and Mor Barak, 2015). For instance, Mor Barak et al. (2001) study the antecedents to retention and turnover among human service employees (including child welfare). DePanfilis and Zlotnik (2008) conducted a systematic review of the literature concerning the retention of frontline employees in child welfare services. Findings from their review pointed toward the importance of several workplace factors implicated in employees’ decision to stay. These factors included employees’ commitment, low levels of emotional exhaustion, self-efficacy, support at work, as well as salary and benefit. Likewise, Kim and Kao (2014) conducted a meta-analytical review of the predictors of turnover intentions among child welfare employees based in the United States. More recently, McFadden et al. (2015) reviewed the child welfare literature focusing on the role of resilience and burnout. The identified themes (subdivided into individual and organizational themes) among the included studies include coping, secondary traumatic stress, social support and supervision, job satisfaction, workload, professional and organizational commitment, etc. As mentioned above, one notable factor here is the heavy reliance/focus on employees’ intentions to stay or leave. Although some of their findings included/associated with workplace support, to our knowledge, workplace support as a concept has been disproportionately left on the sidelines when discussing psychosocial work environment variables and their importance, especially among CWWs.

Given the unequal focus on the negative workplace occurrences among CWWs, does that imply that CWW only deal with negative experiences at work? Au contraire, most scholars would agree that workplace experiences are seldom a one-way street. Against the backdrop of this knowledge, why has the focus on negative outcomes such as turnover, burnout, and occupational stress continue to increase the past years (Kelloway, 2011, p. 1)? One logical explanation could be the notion that “bad is stronger than good.” Baumeister et al. (2001) maintain that negatively valenced events will generate a greater impact on an individual than positive valenced events of the same type. Adding to this is the high economical and emotional cost of having to replace a sick, absent, or a worker who quits. The impact of this to the employer could have generated vibrating attention in the field, influencing the continuous focus on negative occurrences at work. Judging by these factors, it is reasonable that a host of past research centers on turnover and the likes.

## Workplace Support

Before describing the importance of workplace support especially among CWWs, we will briefly present a theory that encapsulates workplace support with other relevant theories in any given work environment. The job-demand resource (JD-R) model provides a more nuanced explanation to what goes on at work. Building on previous balance models of employee well-being [the demand-control model (DCM) by Karasek, 1979; and the effort-reward imbalance (ERI) by Siegrist, 1996], the proponents of the JD-R hold that the exposure of employees to a high demanding work environment coupled with limited autonomy will oftentimes lead to stress and ill health (Bakker and Demerouti, 2007). Furthermore, elevated autonomy will yield a contrasting experience for the employees. The central tenet of this model revolves around two assumed pathways, namely, the health impairment and the motivation processes (Bakker and Demerouti, 2007; Schaufeli and Taris, 2014). The impairment pathway (also referred to as job demands) involves “those physical, social, organizational aspects of the job that require sustained physical or mental effort and are therefore associated with certain physiological and psychological costs” (Demerouti et al., 2001, p. 501). The health impairment could range from job insecurity to role ambiguity, role conflict, unfavorable shift work schedule, and work-home conflict (Schaufeli and Taris, 2014). Job resources or the motivational path centers on those aspects of the job that largely contributes toward the achievement of stated work goals, reduce the impact of job demands and workload, and stimulate growth and development among employees (Schaufeli and Taris, 2014). Examples of job resources are autonomy, advancement, leadership, and social support from both the supervisor and colleagues (Schaufeli and Taris, 2014). The purpose of this scoping review is to assess the importance of workplace support among CWWs.

To get an understanding of workplace support, it is imperative to explore the themes and ideas surrounding it. Workplace support is a sub-arm of social support, which is “the type of assistance that individuals receive from those who come into contact with them in any way” (Papakonstantinou and Papadopoulos, 2010, p. 183). Authors have attempted conceptualizing social support in recent past. This conceptualization varies in focus and content. While some authors focused on categories of support (Huurre et al., 1999), others focus on aspects of social support (Weiss, 1974); elements of support (Birch, 1998; Etzion, 1984), functions of social support (Cohen and Hoberman, 1983), and types of support (Cohen and Wills, 1985; Kef, 1997; Brough and Pears, 2004). Karasek and Theorell (1990) describe workplace support as the sum of support that is available to an individual at the workplace from colleagues and supervisors. In this sense, support could range from receiving advice on a task, appraisals of situations and assignments, information sharing, and emotional support (Karasek and Theorell, 1990; Brough and Pears, 2004; Harris et al., 2007). Past research has shown that workplace support has a positive impact on workplace variables like job satisfaction, engagement, commitment, and negative impact on turnover, burnout, and other negative workplace variables (Karasek and Theorell, 1990; Mor Barak et al., 2001; Brough and Pears, 2004; DePanfilis and Zlotnik, 2008; McFadden et al., 2015). A well-functioning workplace will arguably generate better services to its client than a malfunctioning one. For example, Iversen and Heggen (2016) recently conducted a study on CWWs use of knowledge in their daily work in Norway. Participants reported several factors as vital to performance and quality welfare work. Supervision and colleagues were cited as one of the most important factors. The authors pointed out that “Colleagues and Supervision is not only important for ‘new’ social workers with little experience, but also remains important throughout the career irrespective of working experience and continued education” (Iversen and Heggen, 2016, p. 13).

## The Present Study

In line with the above, the current scoping review will attempt to identify the significance of workplace support among CWW. More importantly, the present review will seek to investigate the relationship (if any) between workplace support and workers experience of psychosocial risks. We endeavor to explore the roles and impact of workplace support among CWW as they carry out their day-to-day tasks at work. The knowledge of this will help researchers, practitioners, and policy makers in focusing on ways to encourage and stimulate support at work thereby influencing the well-being and work environment of CWW. Additionally, we will explore the characteristics of the studies in the field. This review will also identify the gaps in the field, if any.

By investigating and capturing the range of studies exploring the importance of workplace support amidst exposures to psychosocial risks within the child welfare sector, this scoping review will be taking the first step in addressing the gaps in the field of child welfare regarding this theme. Are there any gaps in the field? What areas need more research focus? This present review will attempt to answer these questions regarding the focus of future studies in the field. To our knowledge, no earlier studies have attempted capturing the essence of workplace support among workers in this particular sector.

According to the recently published PRISMA extension for scoping reviews (PRISMA.ScR): checklist and explanation, scoping reviews share numerous components with any other type of knowledge synthesis (Tricco et al., 2018). Scoping reviews have within their scope to “follow a systematic approach to map evidence on a topic and identify main concepts, theories, sources, and knowledge gaps” (Tricco et al., 2018, p. 1). The present review is in line with this relatively new and refined approach to systematic reviews. Using the guidelines as found in the renowned framework of Arksey and O’Malley (2005), this paper aims to employ a systematic and comprehensive exploration of the literature on the workplace support among CWW. This framework has proven to be effective at mapping out the extent, range, and nature of the selected body of research. Our chosen approach is also in line with findings from earlier notable research employing the scoping reviews methodology (Grant and Booth, 2009; Levac et al., 2010; Daudt et al., 2013; Colquhoun et al., 2014; Pham et al., 2014).

Additionally, the current review aims to identify gaps in the literature and provide a summary of results. Specifically, the current paper will employ five key steps as proposed by Arksey and O’Malley (2005): (1) identify the research question, (2) identify relevant studies, (3) study selection, (4) charting the data, (5) collating, summarizing, and reporting the results (Arksey and O’Malley, 2005; Grant and Booth, 2009; Levac et al., 2010; Colquhoun et al., 2014; Pham et al., 2014). As commonly found with scoping reviews, the present study will not make efforts to evaluate the quality of studies nor offer a quantitative synthesis of data. The current scoping review will, however, seek to explore and capture the noteworthy features of an incipient body of evidence. In order to provide a structured overview of the whole research process, we used the Preferred Reporting Items for Systematic Reviews and Meta-Analyses (PRISMA.ScR) guidelines in reporting this scoping review.

## Method

### Stage 1: Identify the Research Question

The present scoping review aims to explore the relationship between workplace support and psychosocial risk exposures among CWW. Following recommendations from Arksey and O’Malley (2005), we intend to begin with a broad review area to establish what is available before narrowing the search. Although past research points toward a negative relationship between workplace support and psychosocial risk, an overview of the types of support, and how essential they are is lacking in the field. Earmarking research questions for this review shapes the focus of the study while directing the identification and selection of relevant studies. The research question: What types of workplace support exist in the literature and what roles do they play in workers outcomes?

### Stage 2: Identify Relevant Studies

#### Search Terms

We implemented the bibliographic databases search from April 15 until 21 September 2018 in PsycINFO, Medline, ProQuest, and the Web of Science. A supplementary search was conducted between the periods of 11 September and 25 September 2019. We selected these databases because they provide a full-bodied coverage of peer-reviewed research and publications on work health and well-being, within which psychosocial risks fall. Since past research is inconclusive regarding how workplace support influences other work environment outcomes, we decided to employ a strategy that considered this knowledge. Using keywords from two widely accepted work environment/psychosocial scales (Copenhagen Psychosocial Scale and QPS-Nordic) as our point of departure, we constructed our search terms and conducted a search in the four aforementioned bibliographic databases. In addition to the two psychosocial scales, we also studied and included notable scales/variables from the PRIMA-EF (Guidance on the European Framework for Psychosocial Risk Management). As pointed out earlier, we were unsure of the range and extent of the publications existing on psychosocial risks among child welfare employees, and therefore we set no limits on publication dates. We employed a simple analytical framework (search, appraisal, synthesis, and analysis—SALSA) as well as two separate Boolean operators “OR” and “AND” in retrieving relevant studies. The first author constructed the search strategy with support from the rest of the team. See Table 1 for the full list of the search terms used in the present study.

**TABLE 1:** Filter for the occurrence of psychosocial risk among child welfare workers.

**Terms relating to risks**	**Child welfare workers**
“Quantitative risks” OR “Cognitive demands” “Emotional demands” “Job demands” OR “Job-strain” OR “Demands for hiding emotions” OR “Sensory demands” OR “Influence at work” OR “Possibility for development” OR “Degree of freedom” OR “Meaning of work” OR “Job commitment” “Predictability” OR “Role clarity” OR “Role conflicts” OR “Conflicts” OR “Leadership quality” OR “supervisor support” OR “Social support” OR “Feedback at work” OR “Social relations” OR “Sense of community” OR “Job insecurity” OR “Job satisfaction” OR “General health” OR “Vitality” OR “Behavioral stress” OR “Vicarious trauma” OR “Somatic stress” OR “Cognitive stress” OR “secondary traumatic stress” OR burnout OR “Sense of coherence” OR “Problem focused coping” OR “Selective coping” OR “Resignation coping” OR “Effort-reward imbalance” OR “Over commitment” OR “Engagement” OR “Turnover” OR “Turnover intentions” OR “intentions to leave” OR “Harassment” OR Bullying OR “Work-life balance”	“Child welfare workers” OR “Child welfare employees” OR “Child welfare professionals” OR “Child welfare social workers” OR “Child welfare employees” OR “child protective service workers” OR “child protective social workers” OR “child protection social workers” OR “Employees within the child welfare” OR “Child protection workers” OR “Child protection professionals”

### Stage 3: Study Selection

The first author conducted the data screening by going through the titles of articles, keywords, and abstracts following broad relevance criteria. We then embarked on a detailed exploration of the full-texts of the chosen articles. After removing duplicates, we identified 2534 citations from searches of electronic databases, highly cited articles, as well as the reference lists of review articles. We screened the titles and abstracts of the 2534 studies and 2386 citations were excluded (because they did not meet the inclusion criteria). Furthermore, we excluded 127 studies that explored psychosocial risk variables in related settings to child welfare, but that was unclear if child welfare employees were included in the sample (e.g. nurses, doctors, teachers, and other social workers), and 155 studies that explored variables outside the scope of the present study (e.g. organizational change, education, proximity to work, etc.). Finally, we excluded two studies because we were unable to retrieve them (we attempted to reach the authors but received no answer). With 148 full text articles remaining to be retrieved and assessed for eligibility, we excluded studies for the following reasons: 52 were dissertations (not pee-reviewed), 37 measured family engagement and related variables, and 14 were not original empirical research (e.g. reports, commentaries, and theoretical studies). We considered 55 studies eligible for this present review.

### Stage 4: Chart the Data

The screening process generated 55 articles that met the inclusion criteria. We developed an extraction spreadsheet in Microsoft Excel in order to maintain a systematic data extraction process. We then moved to Microsoft Word and inserted all of the information from Microsoft Excel into a table. Our coding was guided by the focus of this review. Therefore, we coded included articles by study, sample population, sample size, research design, and psychosocial risk measure and findings.

### Stage 5: Collate, Summarize, and Report Results

According to Arksey and O’Malley (2005), the last stage of the review process entails collating, summarizing, and reporting the results. We achieved this by organizing the relevant results into themes, conscientiously paying attention to these themes as they relate to the research questions and focus of the study.

#### Ongoing Consultation

In order to aid credibility and strength to the review, Arksey and O’Malley (2005) suggest the inclusion of experts in the area of research. For the present review, one professor, two associate professors, and two CWWs with over 12 years of work experience were consulted during the early phase of research questions development, search strategy development, and inclusion criteria. The first author has been involved with two reviews earlier. The fifth author has published more than 10 reviews.

## Results

**FIGURE 1:**
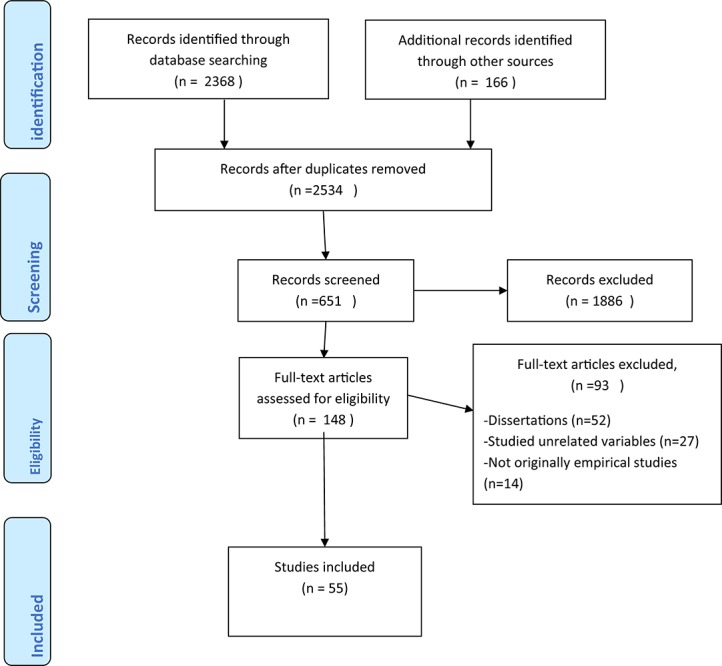
Flow diagram of the study selection.

The studies’ country of origin, design, methods, and key findings are presented in Table 2. Of the 55 included studies in the present review, the majority were from the United States (*n* = 43). Other countries with less than five studies included: Canada, the United Kingdom, Sweden, Australia, Finland, Norway, Israel, and Spain. The vast majority of the included studies were conducted in a single country, except for one study conducted in the United Kingdom, Sweden, and Italy (Frost et al., 2018), and another study conducted in the United States, Finland, the United Kingdom, and Norway (Juhasz and Skivenes, 2018). See Figure 1 for a display of study identification, screening, and the final study selection.

**TABLE 2:** Summary of cohort studies on workplace support among CWW.

**Author and year**	**Sample population**	**Sample size**	**Research design**	**Country**	**Psychosocial work environment measure**	**Key findings**
Aguiniga et al. (2013)	Child protection service	2903	Quantitative	United States	Intention to leave, positive mood about work, collegiate support	Positive mood about work and collegiate support both predicted intention to leave. Gender, race, and age all significant predictors of workers’ intention to leave
Antonopoulou et al. (2017)	Child protection service	193	Quantitative	United Kingdom	Psychological distress and anxiety, work enabling conditions, social support at work, job autonomy, and decision-making	Low levels of stress reported. Reports of positive association between stress and job clarity, control, as well as management and social support at work
Augsberger et al. (2012)	Voluntary public child welfare	538	Mixed methods	United States	Turnover, organizational support, fair salary and benefits, fair promotion potential, adequate communication, and appreciation	Workers perceptions of respect in the workplace predict turnover intentions. This is further broken down into five sub-themes of organizational support, fair salary and benefits, fair promotion potential, adequate communication, and appreciation or contingent rewards
Baldschun et al. (2016)	Child protection social workers	364	Quantitative	Finland	Well-being (affective, cognitive, social, personal, professional, and psychosomatic), supportive work environment	Findings suggest that affective well-being as well as an open and supportive work environment is crucial to any occupational well-being of employees
Barak et al. (2006)	Child welfare workers	418	Mixed methods	United States	Perceptions of fairness, inclusion–exclusion, social support, stress, well-being, commitment, job satisfaction, and intentions to leave	Findings suggest that stressful, unfair, non-inclusive/supportive organizational climate, as well as various individual characteristics negatively influence employees’ well-being. This leads to job dissatisfaction and lower commitment, which further lead to employees’ intentions to leave the organization
Barbee et al. (2009)	Child welfare workers	15	Qualitative	United States	Turnover correlates, supervision, colleagues support	Respondents reported that the search for a more lucrative position, lack of supervision, stress, and the lack of support from colleagues were some of the reasons they left their earlier jobs
Baugerud et al. (2018)	Child protection workers	506	Quantitative	Norway	Quantitative demands, role expectations, control over work intensity, stress, predictability of work, burnout, social interaction and support, work-life balance, secondary traumatic stress, compassion satisfaction	Findings showed the prevalence of moderate symptoms level of stress-related issues of burnout and secondary traumatic stress. Furthermore, respondents equally reported a moderate compassion satisfaction level. Findings are contrary to results from earlier studies
Biggart et al. (2017)	Child and family social workers	52	Qualitative	United Kingdom	Emotional experiences, team physical and work environment, supervision, and information support	Reports of reflective supervision, socio-affective needs, and sharing of emotional experiences with colleagues
Bride (2007)	Protective service workers	187	Quantitative	United States	Secondary traumatic stress, peer support, administrative support, turnover intention, professional experience, and workload	Secondary traumatic stress was associated with workers’ personal trauma history, peer support, administrative support, turnover intention, professional experience, and workload.
Chen and Scannapieco (2010)	Protective service workers	453	Quantitative	United States	Turnover, self-efficacy, supervisor support, job satisfaction	Main effect of job satisfaction, supervisor support, and self-efficacy on employee’s turnover intentions
Chenot et al. (2009)	Public child welfare workers	767	Quantitative	United States	Organizational culture, supervisor support, turnover intention, retention	Longevity decisions are mostly important the first 3 years of service. Supervisor and peer support both predicted retention. Supervisor support had a stronger effect than peer support, and supervisor support effects cut across the entire samples
Cohen-Callow et al. (2009)	Child welfare workers	561	Quantitative	United States	Career commitment, climate, commitment, supervisor/coworker support, job withdrawal, job satisfaction, stress	Reports of lower job and work withdrawal by older respondents. The association between withdrawal and commitment varied across age. Experience of stress best predicts job and work withdrawal
Csiernik et al. (2010)	Child protection workers	13	Mixed methods	Canada	Hope, social support, anxiety levels associated with clients, resilience	Findings show the prevalence of anxiety toward clients, the work group, as well as employees’ own ability. Reports of various types of stressful incidents. Respondents also reports experiences of resilience and social support
Curry et al. (2005)	Protective service workers	441	Quantitative	United States	Case load, supervisor support, retention	Experience, gender, and education were associated with staff retention. Supervisor support and the overall transfer potential, as well as application planning transfer were all positively associated with transfer
Dagan et al. (2016)	Child protection workers	124	Quantitative	Israel	Secondary traumatic stress, mastery, social support, effectiveness of supervision, role stress, traumatic experiences	Reports of high level of secondary traumatic experiences. Findings also show that role stress contributed significantly to secondary traumatic experiences. A positive association was also found between employees’ experiences and secondary traumatization
Davis-Sacks et al. (1985)	Child welfare workers	62	Quantitative	United States	Supervisor support, coworker support, spouse support, mental health problems, burnout	Most of the respondents report experiencing support from spouse, coworker, and supervisors. Except self-esteem, coworker support is not significantly associated with burnout and mental health problems
Dickinson and Perry (2002)	Public child welfare workers	235	Quantitative	United States	Job roles, responsibilities, caseload, job satisfaction, social/supervisory support, turnover intentions, work conditions, burnout, stress	Respondents who intend to leave or already left cited several reasons for this decision. Stress, dissatisfaction with the work environment, changes in career goals, and availability of other jobs were the four most important reasons
Fernandes (2016)	Child welfare workers	359	Quantitative	United States	Turnover intention, organizational support, workload	Perceptions of organizational justice, organizational support, workload, and job importance predicted workers’ turnover intentions
Festinger and Baker (2010)	Child welfare personnel	253	Quantitative	United States	Childhood trauma, self-esteem, satisfaction with life, sense of social support	Significant associations were found between experienced childhood emotional maltreatment and the three well-being measures (i.e. self-esteem, satisfaction with life, and sense of social support). Findings showed a prevalence of 30% reported rate of recall for childhood emotional abuse. Emotional abuse report was more prominent among female respondents
Fryer et al. (1989)	Child protection workers	300	Quantitative	United States	Attrition, intentions to leave, coworker support, stress, anxiety, commitment	Findings show high reports of both stress and coworker support. The professional aspects of child protection were positively associated with work commitment. Intentions to leave were also associated with reports of resentment, anxiety, helplessness, and regrets for joining the field
Fryer et al. (1988)	Child protection workers	301	Quantitative	United States	Workload, compensation, attitudes to work, coworker support, job dissatisfaction	Findings show reports of high workload and effort-reward imbalance. Respondents also report high coworker support. Work experience with child welfare service is positively associated with job dissatisfaction
Griffiths and Royse (2017)	Former CWS workers	54	Mixed methods	United States	Turnover, job dissatisfaction, work experience, workload, lack of respect, organizational support	Job dissatisfaction, work experience, workload, lack of respect, organizational support was all associated with turnover
He et al. (2018)	Child welfare workers	1917	Quantitative	United States	Burnout, demands, resources, supervision, and peer support, workload	Job demands, supervision, and peer support were positively associated with client-related burnout
Healy et al. (2009)	Child welfare workers	58	Qualitative	Australia	Turnover, stress, effort-reward imbalance, support	Reports of high inexperience coupled with high workload. Experienced employees not motivated to go into the field. High stress contributing to turnover. Absence of support and developmental opportunities. Reports of a blaming culture, low reward, and lack of respect for staffs
Hermon and Chahla (2019)	Child welfare workers	160	Quantitative	United States	Stress, child-related stress, visit-related stress, workload stress, satisfaction, client relationships, work-life flexibility, growth and support, perceptions of caseload, turnover, retention	Findings showed that the experience of high level of stress predicts turnover among employees. Results also showed that job stress has damaging effects on the stayers
Hunt et al. (2016)	Child protection workers	423	Quantitative	United Kingdom	Violence, support, supervision	Respondents reported experiencing threats or violence from parents. Some respondents also reported not receiving sufficient support and supervision from the management. Making situations worse as opposed to bettering them
Jacquet et al. (2008)	Fresh CWS graduates	765	Quantitative	United States	Turnover intention, Supervisor support	Supervisor support was strongly associated with workers intent to leave
Jayaratne et al. (1986)	Child welfare workers	238	Quantitative	United States	Work stress, strain, emotional support, mental health, job satisfaction, burnout	Findings suggest that employees reported high rate of burnout also scored higher on mental health issues, lower on marital satisfaction, and somatic complaints. Reports of burnout were also significantly (negative) associated with perception of support both from supervisors and colleagues
Juhasz and Skivenes (2018)	Child welfare workers	474	Vignette experiment	United States, Finland, England, Norway	Caseloads, management/organizational factors, cooperation factors	Time/caseloads factors were the most common among respondents across countries. Respondents also reported experiencing management/organizational constraints regarding proper decision-making. Other cited constrains are threshold/evidence issues and cooperation with other bodies
Juby and Scannapieco (2007)	Child welfare workers	350	Quantitative	United States	Workload satisfaction, support, resources, worker ability	Availability of resources was found to be the most influencing variable. Availability of resources was associated with workload satisfaction and worker ability. Supervisor support was also associated with workload satisfaction
Kim and Hopkins (2015)	Child welfare workers	435	Quantitative	United States	Organizational commitment, safety concerns and unsafe climate, coworker support, leader–member exchange, role conflict, and clarity	Findings showed that unsafe organizational climate was negatively associated with organizational commitment. This relationship is further strengthened among employees who experienced low quality of LMX
Kim et al. (2018)	Child welfare workers	1244	Quantitative	United States	Supervisory support, worker’s role, work experience, workload	Findings show that the perception of supervisory support significantly decreased up until the 12 years work experience mark; and it significantly changes course after this period maintaining a curve linear with a U-shaped curve. Results support the assumption that frontline workers have varying needs of support according to their developmental stages
Kruzich et al. (2014)	Public child welfare workers	1040	Quantitative	United States	Perceived organizational support, perceived supervisor support, team psychological safety, intentions to stay	Findings showed that both human resource primacy, as well as empowering and supportive leadership style, influenced employees’ stays intentions through the mediating role of psychological safety
Landsman (2001)	Child welfare workers	1133	Quantitative	United States	Turnover intention, job stressors, perceived agency support, job satisfaction, organizational commitment, workload	Perceived agency support is associated with organizational commitment. Workload has significant effect on organizational commitment, job satisfaction, and turnover intention
Landsman (2008)	Public child welfare workers	497	Quantitative	United States	Organizational commitment, Service orientation, job safety, role ambiguity, distributive justice, supervisor support	Service orientation, job safety, role ambiguity, distributive justice, supervisor support, were all associated with workers commitment
Lee et al. (2017)	Child welfare workers	104 51	Mixed methods	United States	Coping strategies, work stress, supervisor support, caseload	Findings showed reports of high caseload and lack of time, spillover of work stress to family life, and lack of supervisor support and disregard for child welfare workers’ self-care needs. Additionally, respondents reported the use of negative coping strategies like alcohol, drugs, and denial
Littlechild et al. (2016)	Child welfare workers	590	Mixed methods	United Kingdom	Aggression, violence, work-life balance, roles, support at work, anxiety	Findings showed that respondents experience plethora of violence and aggression resulting into anxiety, depression, disturbed sleep, sleeplessness, and panic attacks. Others reported that they have been forced to change addresses, cars and names. Several (38%) of the respondents also reported insufficient support at work
Lizano and Barak (2012)	Public child welfare workers	335	Quantitative	United States	Job stress, work-family conflict, emotional exhaustion, organizational support	Organizational tenure, job stress, and work-family conflict were associated with emotional exhaustion development. Age, work-family conflict, and organizational support were related to the development of depersonalization
Lovseth (2017)	Child welfare workers	142	Quantitative	Norway	Confidentiality as a barrier for support, coping, proximity to clients	Findings suggests that clients confidentiality can influence the child welfare employee’s personal support system. Results also showed a marked difference between experienced child welfare employees and the ones that are relatively new to the workforce
Madden et al. (2014)	Public child welfare workers	9195	Quantitative	United States	Turnover, retention, organizational support	Gender, social work education, position, organizational support, and job desirability were found to significantly influence workers’ decisions to stay on the job
Barak et al. (2006)	Child welfare workers	418	Mixed methods	United States	Perceptions of fairness, social support, inclusion–exclusion, organizational stress, well-being, organizational commitment, job satisfaction, turnover intentions	Findings showed that job satisfaction, low organizational commitment, younger age, high stress and exclusion from the organizational decision-making processes were all the strongest predictors of turnover. Furthermore, results suggested that experiencing a stressful, unjust, exclusionary and non-supportive organizational climate, with various individual characteristics could negatively influence employees’ well-being and job satisfaction. This can further lead to stronger turnover intentions
Morazes et al. (2010)	Child welfare social workers	386	Qualitative	United States	Retention, workload, support from colleagues	Findings showed that respondents have trouble with heavy workloads, time pressure, and hindrances from carrying out “true social work functions.” Additionally, there were marked differences between the stayers and the leavers in the level of reported support from colleagues
O’Donnell and Kirkner (2009)	Public child welfare workers	267	Quantitative	United States	Burnout, role conflict, supervision, commitment, job satisfaction, supervisor support, retention	Demographics variables were not significantly associated with employees’ retention. Measuring retention in two separate years shows a variance in the influence of factors like commitment, role conflict, burnout, supervisor support, and job satisfaction
Radey et al. (2018)	Child welfare workers	38	Qualitative	United States	Support	Findings showed that employees experience support in its type (instrumental or expressive) and source (family, coworker, supervisor, and friends)
Salloum et al. (2015)	Child welfare workers	104	Quantitative	United States	Burnout, secondary trauma, compassion satisfaction	Trauma-informed self-care was associated with higher levels of compassion satisfaction and lower levels of burnout
Salloum et al. (2018)	Child welfare workers	177	Quantitative	United States	Compassion satisfaction, secondary traumatic stress, burnout, psychological well-being, support	Utilizing organizational resources, organizational practices, and professional self-care were all negatively associated with secondary traumatic stress and burnout. Additionally, positively associated with psychological well-being, compassion satisfaction, and organizational resources provided
Scannapieco and Connell-Carrick (2007)	Child welfare workers (former and current)	1283/598	Quantitative	United States	Training, turnover, support	The two groups are similar in work experience, assessment of physical and sexual abuse. Transition to practice, training, and supervisor support were associated with turnover
Schelbe et al. (2017)	Child welfare workers	38	Qualitative	United States	Stressors, unsupportive colleagues, workload, job satisfaction	Workers reported high job satisfaction. They also reported stressors in through; administrative requirements, workload, unsupportive colleagues, and challenging clients
Smith (2005)	Child welfare workers	296	Quantitative	United States	Work-life balance, perceived organizational support, job clarity, job commitment, retention	Employees who considered quitting at Time 1 were more likely to have done so by Time 2. Supervisor support and organizational level variables were found to be associated with employees’ retention
Tham and Meagher (2009)	Child welfare workers	309	Quantitative	Sweden	Demands, role clarity, role conflict, support, social climate	Reports of high job demands and greater control regarding decision-making. Additionally, high role conflict and less role clarity than the comparison group
Travis and Mor Barak (2010)	Child welfare workers	359	Quantitative	United States	Role conflict, stress, and ambiguity	Workers differed in their voice, neglect, and exit responses by gender, ethnicity, job level, and job tenure. Neglect and exit were positively related to role stress. Workers with high sense of psychological well-being were less likely to report exit- and neglect-related efforts
Westbrook et al. (2006)	Public child welfare workers	21	Qualitative	United States	Personal and organizational factors involved in employees retention, supervision and support, commitment	Findings point toward the importance of establishing an organizational climate and culture that prioritizes the care of children and families in need. Close monitoring, supervision, and support will boost new employees’ commitment. Recognition of hard work was also found to increase retention among employees
Williams et al. (2011)	Public child welfare workers	260	Mixed methods	United States	Turnover, coworker support, reasonable workloads, opportunities for advancement, supervisor support, valuing employees, and organizational commitment	Better salaries, coworker support, reasonable workloads, opportunities for advancement, supervisor support, valuing employees, and organizational commitment were all associated with turnover
Wu et al. (2013)	Public child welfare workers	573	Quantitative	United States	Work-life balance, Organizational support	Organizational support, job value, work time, and income were all associated with work-life balance
Yankeelov et al. (2009)	Protective service workers	723	Quantitative	United States	Turnover, retention, social provisions, supervisor support, workplace environment,	Turnover estimates range from 14 to 36% for first to third years, respectively. Stayers and leavers do not differ in race and gender

Support as framed by these studies somewhat varied, although they all shared similar scope. Depending on the study in focus, “support” was framed as colleagues/collegiate support, organizational support, social support, peer support, administrative support, supervisor support, and coworker support. Others framed support as emotional support, perceived agency support, and perceived organizational support. Although the included studies framed support differently, a closer look shows that they all could be categorized under three main support types of coworker/peer support, social/organizational/management support, and supervisor/leadership support. These three are presented below.

### Coworker/Peer Workplace Support

Eighteen of the included studies investigated the impact of peer support among CWW (Davis-Sacks et al., 1985; Jayaratne et al., 1986; Fryer et al., 1988, 1989; Landsman, 2001; Bride, 2007; Barbee et al., 2009; Chenot et al., 2009; Cohen-Callow et al., 2009; Morazes et al., 2010; Williams et al., 2011; Aguiniga et al., 2013; Salloum et al., 2015; Biggart et al., 2017; Schelbe et al., 2017; Baugerud et al., 2018; He et al., 2018; Radey et al., 2018; Hermon and Chahla, 2019). Jayaratne et al. (1986) found a negative relationship between coworker support and reports of burnout. The authors further conducted a series of T-tests to compare the highs and the lows of support scores. Only two out of the nine tests were significant, coworker support being one of them. Bride (2007) report a negative (significant) correlation between workers’ experience of secondary traumatic stress and peer support. They concluded that employers could reduce workers experiences of secondary traumatic stress by cultivating opportunities for peer support. In their study on the examination of the internal and external resources in child welfare, He et al. (2018) found that peer support was negatively associated with client-related burnout. Likewise, in their study of survival among recently hired CWW, Radey et al. (2018) found that CWWs place a very high value on “expressive support” from colleagues. They reported one of the respondents saying “I’m surrounded by a lot of caring individuals who not only care about the kids and the families that they are helping, but their team members” (Radey et al., 2018, p. 89). Other studies found peer support to reduce experiences of stress, burnout, turnover, while serving as a source of strength and increasing retention (Davis-Sacks et al., 1985; Fryer et al., 1988, Fryer et al., 1989; Barbee et al., 2009; Chenot et al., 2009; Morazes et al., 2010; Williams et al., 2011; Biggart et al., 2017; Schelbe et al., 2017).

### Social/Organizational/Management Workplace Support

Twenty-two of the included studies explored themes related to CWW experiences and/or consequences of organizational or management support (Samantrai, 1992; Landsman, 2001; Smith, 2005; Barak et al., 2006; Healy et al., 2009; Csiernik et al., 2010; Travis and Mor Barak, 2010; Augsberger et al., 2012; Lizano and Barak, 2012; Wu et al., 2013; Kruzich et al., 2014; Madden et al., 2014; Salloum et al., 2015; Baldschun et al., 2016; Dagan et al., 2016; Fernandes, 2016; Hunt et al., 2016; Littlechild et al., 2016; Antonopoulou et al., 2017; Griffiths and Royse, 2017; Hermon and Chahla, 2019). Healy et al. (2009) conducted an international comparative study comprising of respondents from Australia, the United Kingdom, and Sweden. They found work stress, low reward, a culture of blame, and lack of support implicated in workers’ retention. In her study of commitment among CWW, Landsman (2001) found that the experience of low commitment is one of the major predictors of turnover. The study also found agency/management support to be one of the predictors of work commitment. Management support was also positively related to job satisfaction, but this relationship was not significant. Likewise, Lizano and Barak (2012) employed the JD-R model to explore the impact of organizational support on burnout among CWW. The authors found a positive and significant relationship between workers’ perception of organizational support and depersonalization (one of the three sub-categories of burnout). Meaning that less reports of support was related to more burnout (depersonalization). Eight studies investigated the influence of management support on retention and turnover (Samantrai, 1992; Smith, 2005; Travis and Mor Barak, 2010; Augsberger et al., 2012; Kruzich et al., 2014; Madden et al., 2014; Fernandes, 2016; Griffiths and Royse, 2017). Perception of organizational support influences workers’ decisions to stay/leave. This finding was common for all studies. Wu et al. (2013) explored the essence of organizational support for workers’ work-life balance. They reported a positive relationship between workers perceived organizational support and experiences of a higher work-life balance. Both Hunt et al. (2016) and Littlechild et al. (2016) investigated the importance of organizational support among CWW facing violence and aggression from parents. In some of the included studies, participants reported different sets of safety measures installed in order to keep threats to their lives at bay. Some of these measures include necessity to change their real names, cars, fitting alarms to their homes, and other types of surveillance systems. Participants also reported experiencing anxiety, panic attacks, inability to sleep, and taking time off from work. Some of these workers reported that they did not get any support not understanding from the management despite the seriousness of their circumstances working with aggressive and violent clients (Littlechild et al., 2016). Other studies reported that social support was negatively and significantly correlated with secondary traumatization among a sample of CWW (Salloum et al., 2015; Dagan et al., 2016).

### Supervisor/Leadership Workplace Support

Twenty-three of the included studies explored supervisor support among CWWs (Davis-Sacks et al., 1985; Jayaratne et al., 1986; Samantrai, 1992; Dickinson and Perry, 2002; Curry et al., 2005; Smith, 2005; Westbrook et al., 2006; Juby and Scannapieco, 2007; Scannapieco and Connell-Carrick, 2007; Jacquet et al., 2008; Landsman, 2008; Chenot et al., 2009; Cohen-Callow et al., 2009; O’Donnell and Kirkner, 2009; Yankeelov et al., 2009; Chen and Scannapieco, 2010; Williams et al., 2011; Kruzich et al., 2014; Littlechild et al., 2016; Lee et al., 2017; Kim et al., 2018; Radey et al., 2018; Hermon and Chahla, 2019). One study looked at the relationship between supervisor support and stress, as well as the relationship between supervisor support and satisfaction (Hermon and Chahla, 2019). Using a series of multilevel models, Chenot et al. (2009) investigated the influence of supervisor support among newly recruited CWW. They found that supervisor support predicted retention among newly recruited CWW. Thirteen other studies examined supervisor support and retaining workers approaching retirement (Cohen-Callow et al., 2009), retention (Dickinson and Perry, 2002; Smith, 2005; Scannapieco and Connell-Carrick, 2007; Jacquet et al., 2008; O’Donnell and Kirkner, 2009; Yankeelov et al., 2009; Williams et al., 2011), turnover (Curry et al., 2005; Kruzich et al., 2014), violence and aggression against CWW (Littlechild et al., 2016), desire to stay, and decision to leave (Samantrai, 1992; Chen and Scannapieco, 2010).

## Discussion

In this scoping review, we identified 55 primary studies exploring a plethora of workplace support related themes among CWW published between 1985 and 2019. The included studies focused on different types of support at work, they also explored the relationship between their chosen workplace support and job satisfaction, job commitment and engagement, secondary traumatic stress, violence, turnover intentions, intentions to stay, retention, and turnover. They also investigated the roles of support at work in heavy and high workload, job stress, burnout, secondary traumatic stress, vicarious trauma, role stress, role conflict, role ambiguity, career advancement, unmet expectation, work-family conflict, work-life balance, and emotional exhaustion.

### Essence of Workplace Support

Our findings show that the essence of workplace support is found in its dual ability, i.e. the potential to increase positive workplace outcomes (e.g. job satisfaction, engagement, commitment, meaning in the work) while simultaneously reducing the likelihood of occurrence or the effect of exposure to negative workplace experiences (e.g. burnout, stress, trauma, heavy workload). It is logical to think that workers who experience support by the management, supervisors, and colleagues are more likely to feel more secured, happy with their work, experience a very sense of belonging and positive attachment to the workplace, and are also more likely to exert substantial efforts toward any given tasks. Concerning the results from the current review, that workplace support ameliorates CWW’s negative experiences at work, this is in line with recent findings from reviews (Mor Barak et al., 2001; DePanfilis and Zlotnik, 2008). As mentioned in Section “Introduction,” workplace support was implicated in workers decision regarding staying or quitting their positions (DePanfilis and Zlotnik, 2008). The stayers reported experiencing more support than the leavers did.

Similarly, the study conducted by McFadden et al. (2015) investigated individual and organizational factors associated with resilience and burnout among CWW. Having peer support and supervisor support provided a buffering effect on burnout and turnover. One could also argue that initiating and practicing workplace support is cost effective in the sense that the giving and experiencing of support does not have to cost anything in terms of resources. Just that path on the back, the gentle look of compassion and understanding when a coworker or subordinate complains about their experiences will go a long way in helping these coworkers deal with the exposures to negative workplace outcomes. It is almost like the saying “the best things in life are free,” providing and receiving support as shown by the included studies portrays an action or activity that takes so little to achieve but yet generates a significant effect when received. Past research on thriving at work and psychological safety also provides support for the importance of workplace support (Kahn, 1990: Paterson et al., 2014).

The essence of workplace support is also made obvious especially in its absence. In one of their studies on aggression and violence against CWW, Littlechild et al. (2016) reported one of the respondents mentioning that she repeatedly received death threats and threats of violence, with her home address available to these potential assailants, and that it took a while before the management took her seriously. It is common that workers put in a lot of efforts into what they do at work and things get too overpowering as a result of the emotions involved. This could be related to the difficult decisions they have to make, or the outright weight of the amount of cases they have to deal with, it is safe to argue that having the understanding and support of the management, supervisor, and colleagues becomes necessary in order to appropriately deal with these risks. In their definition of social support in the workplace, Karasek and Theorell (1990) did not mention the roles of the management. The support of the management is quite important to any CWW. Added to this is that most CWWs agencies could have up to three levels of leadership positions (the main leader, section leader, and the workgroup leader). In view of this, we want to define workplace support as the sum of support available to a worker at her workplace. And this includes support from the management, supervisor/leader, as well as from colleagues and every notable bodies/groups in the agency.

While results from this review showed a high focus on numerous workplace outcomes as they relate with workplace support among CWW, our findings also indicated a paucity of research focusing on other essential areas of work environment. For instance, we rarely find studies investigating themes like harassment, bullying, organizational citizenship behavior (OCB), and counterproductive work behavior (CWB). These workplace variables are particularly essential because of the roles they play in employees’ day-to-day experiences at work, as well as employees’ well-being and health. Looking at the literature, especially within sectors outside child welfare, past research has linked variables like employees experiences of bullying, workplace harassment, OCB, and CWB with employees well-being, health, and performance (Smith et al., 1983; Organ, 1997; Podsakoff et al., 2000; Rotundo and Sackett, 2002; Dalal, 2005; Podsakoff et al., 2010). Take OCB and CWB as examples, their proponents argue that social exchange theory–(Thibaut and Kelley, 1959, as cited in Arya and Kaushik (2015); the theory of psychological contracts–(Rousseau, 1989); and the norm of reciprocity—(Gouldner, 1960) explains occurrence of OCB and CWB on the one hand and job satisfaction, experience of organizational justice, and organizational commitment on the other (Dalal, 2005). The crucial point here is that the working conditions of CWW should be taken seriously as the rest of the other employees found in any other sector. Owing to the nature of their work, expectations from parents and society, and the difficulties they experience daily, CWW’s health and well-being within the workspace deserves in our opinion more attention than the status.

As pointed out in Section “Introduction,” CWW can be compared to employees within SCOs where decisions, especially wrong ones, could have damaging consequences to those involved and the society. A critical look at the workplace experiences of employees from the SCOs within Norway, for instance, illustrates largely a different picture compared with the child welfare service. Under the umbrella of the petroleum safety authority Norway (PSA), the industry conducts annual and biannual investigations of the work environment, and especially as they relate to themes like safety, which is a core theme to all and sundry in this sector. According to PSA (2019), conducting annual/biannual studies and safety work environment investigations increases awareness of the specific HSE challenges facing the industry, and this in turn enables them to conduct effective preventive work environment and safety work (PSA, 2019). In a similar fashion, the existing state of affairs within the child welfare regarding workplace and work conditions begs for focus on the core workplace environmental variables. We believe attention should be directed toward variables like workload, bullying, harassment, organizational justice, OCB, CWB, etc., not only because past research has shown their importance, but more so because “there is a certain, poetry in behaving badly in response to some perceived injustice” (Sackett and DeVore, 2001, p. 160).

Put together, our findings showed that the majority of the included studies focused largely on turnover (or related themes, i.e. turnover intentions, stay intentions, and retention). At this junction, one can safely say that we now have a working knowledge of these variables. Thus, research that focus on a comprehensive/all-inclusive approach will go a long way to providing us with tools needed to better the work environment of employees within the child welfare. While the focus on these aforementioned themes are not inherently wrong nor far-fetched (there are abundance of useful knowledge from findings reported by past studies), we believe that shifting the focus (or expanding the focus) to cover those crucial areas might enable us to know more about a healthy work environment among this workgroup. The argument here is that authors should embrace focusing on factors that stimulate a better working culture, successful independent and collective working groups, as well as a healthy work environment, as opposed to concentrating heavily, on why employees are leaving or thinking about leaving. It is plausible to argue that this line of research will eventually safeguard against the reliance on last minute or what we can refer to as the fire brigade approach to research on turnover, turnover intentions and retentions among CWW.

### Dealing With the Fire Brigade Approach

The “fire brigade approach” refers to desperate actions taken to quench a deteriorating situation, as opposed to earlier devotions/interventions to its causes. Our findings identified a host of psychosocial risk factors that are associated with workplace support. Remaining in a workplace/workforce does not always translate into thriving and good experience. Research on absenteeism and presentism has shown that employees could remain on the job while constantly calling in sick. This will eventually add more workloads to the rest of their workgroup. Employees may also show up every working day without putting in any tangible efforts or contributions toward the expected task owing to sickness and indisposition (Jensen and McIntosh, 2007). Authors refer to this as presenteeism or disengagement (Aronsson and Gustafsson, 2005; Hansen and Andersen, 2008). When we measure a functional child welfare workplace in terms of those who stay or leave, we miss these groups of employees that are sick or just not doing their jobs.

### Strengths and Limitations of the Present Review

Since this is the first paper to focus on the importance of workplace support among CWW (to our knowledge), this will provide scholars and policy makers with the state of events in the field especially as regarding the importance of workplace support. The inclusion of only peer-reviewed articles (scientific evidence) adds more credibility to the study. The present scoping review has some limitations. The majority of the included studies were published in the United States. Another limitation common to all scoping reviews is the absence of a quality assessment of the included studies. We must, however, point it out that scoping reviews are conducted to identify research in a given field, and quality assessment falls outside of its scope and focus (Arksey and O’Malley, 2005). Owing to the aims of our study, we streamlined our search to focus only on studies exploring the importance of workplace support among child welfare employees. Although our strategy appears to be clear, but we might have missed some studies even though they included CWWs in their sample. This may have occurred if studies have been unclear about the composition of their sample data.

### Further Research Opportunities

– The influence of leadership styles on CWWs’ exposure to and coping with psychosocial risk at work.
– The role of a health promoting work climate and culture on performance, health, and well-being among CWWs.
– The prevalence and impact of harassment, violence, and bullying among child welfare employees, and their effect on performance and well-being.
– The connection between employees’ exposure to psychosocial risk and deplorable workplace behaviors.

## Conclusion

In view of the impacts of psychosocial risks on workers’ health and well-being, efforts should be geared toward raising awareness on the importance of support among CWW, especially as it relates to the risks identified in the present scoping review. Research on the association between workplace support and psychosocial risks among CWW is not new, but this review is the first to summarize and analyze existing literature on the essence of workplace support among CWWs.

## Author Contributions

All listed authors have contributed substantially to the final manuscript and approved it for publication.

## Conflict of Interest

The authors declare that the research was conducted in the absence of any commercial or financial relationships that could be construed as a potential conflict of interest.
